# Citizen Science Program Shows Urban Areas Have Lower Occurrence of Frog Species, but Not Accelerated Declines

**DOI:** 10.1371/journal.pone.0140973

**Published:** 2015-11-18

**Authors:** Martin J. Westgate, Ben C. Scheele, Karen Ikin, Anke Maria Hoefer, R. Matthew Beaty, Murray Evans, Will Osborne, David Hunter, Laura Rayner, Don A. Driscoll

**Affiliations:** 1 Fenner School of Environment and Society, The Australian National University, Canberra, ACT, 2601, Australia; 2 ARC Centre of Excellence for Environmental Decisions, The Australian National University, Canberra, ACT, 2601, Australia; 3 ACT and Region Frogwatch, Ginninderra Catchment Group, Canberra, ACT, 2615, Australia; 4 CSIRO Land and Water, Canberra, ACT, 2601, Australia; 5 Environment and Planning Directorate, ACT Government, Canberra, ACT, 2601, Australia; 6 Institute for Applied Ecology, University of Canberra, Canberra, ACT, 2601, Australia; 7 NSW Office of Environment and Heritage, Queanbeyan, NSW, 2620, Australia; 8 School of Life and Environmental Sciences, Centre for Integrative Ecology, Deakin University, Melbourne Burwood Campus, Burwood, VIC, 3125, Australia; Universität Zurich, SWITZERLAND

## Abstract

Understanding the influence of landscape change on animal populations is critical to inform biodiversity conservation efforts. A particularly important goal is to understand how urban density affects the persistence of animal populations through time, and how these impacts can be mediated by habitat provision; but data on this question are limited for some taxa. Here, we use data from a citizen science monitoring program to investigate the effect of urbanization on patterns of frog species richness and occurrence over 13 years. Sites surrounded by a high proportion of bare ground (a proxy for urbanization) had consistently lower frog occurrence, but we found no evidence that declines were restricted to urban areas. Instead, several frog species showed declines in rural wetlands with low-quality habitat. Our analysis shows that urban wetlands had low but stable species richness; but also that population trajectories are strongly influenced by vegetation provision in both the riparian zone and the wider landscape. Future increases in the extent of urban environments in our study area are likely to negatively impact populations of several frog species. However, existing urban areas are unlikely to lose further frog species in the medium term. We recommend that landscape planning and management focus on the conservation and restoration of rural wetlands to arrest current declines, and the revegetation of urban wetlands to facilitate the re-expansion of urban-sensitive species.

## Introduction

The majority of the human population now lives in urban areas [1], and the total area devoted to urban land uses continues to rise in many regions of the world [2]. Although this process is considered to be broadly detrimental to biodiversity [3,4], urban areas are capable of sustaining diverse plant and animal assemblages in many instances [5,6]. Further, biodiverse cityscapes provide a range of ancillary benefits, including greater integration with water-sensitive urban design [7], mitigation of urban heat island effects [8], and improved human well-being through greater association and engagement with nature [9]. While maintaining diverse plant communities may be achieved through preservation or revegetation, conservation of urban animal populations is much more challenging. Therefore, a valuable goal for ecology is to identify circumstances where landscape planning can be used to facilitate the persistence of threatened or valued animal species in urban and peri-urban areas. This goal requires identification of attributes of those areas (such as the quality and availability of suitable habitats) that are associated with declines or expansions of biodiversity.

While the study of urban biodiversity has expanded significantly in recent years [5], considerable gaps remain in our knowledge of how urbanization drives ecosystem change. As such, it is often unclear how new and existing urban environments can be planned and managed to facilitate the persistence of biodiversity. For example, studies of urban bird populations have shown that retention of keystone structures such as large old trees can have a strong, positive effect on suburban biodiversity [10], but this effect is also dependent on urban density [11]. Further, some threatened species respond negatively to the rate of expansion of urbanization, irrespective of whether their home range is directly impacted [12]. These studies highlight that urbanization has a range of correlated impacts on animal populations, meaning that it can be difficult to predict which management or planning activities will be of greatest benefit to biodiversity. They also suggest that piecemeal changes to urban density, the rate of urban expansion, or the attributes of urban parks and gardens are unlikely to be effective at retaining biodiversity in urban locations when implemented in isolation [11].

A further reason it is difficult to quantify the effects of urban planning on biodiversity is that these effects can manifest over several years or even decades, a time-scale for which comprehensive survey data are rarely available [13,14]. Consequently, studies of the effects of urbanization typically involve short-term studies of differences in biotic assemblages across an urbanization gradient [15], or between towns or cities of differing age [16]. While these approaches provide valuable results in many cases, space-for-time substitution can confound site and treatment effects, and does not allow the trends of individual populations to be observed through time [17,18]. An effective alternative is to use long-term datasets collected through citizen science initiatives, which often provide much higher spatial and temporal replication than expert surveys at lower cost [19,20]. These datasets have potentially enormous utility for investigating questions about the patterns and drivers of biotic change [21], provided that potential sampling biases are addressed in a rigorous statistical framework [22]. Citizen monitoring of plant or animal populations is particularly valuable in urban areas where volunteers (and therefore records) are proportionally more common [23,24].

In this paper, we use a 13-year citizen science dataset to investigate how urbanization influences the occurrence or trajectory of frog populations, and the extent to which these effects are altered by vegetation structure at (or around) wetland habitats. Quantifying trends in urban frog populations is important because changes in the type and intensity of human land use represent one of the most significant threats to amphibian biodiversity [25]. Further, while the effect of urbanization on frog populations is generally thought to be negative [26], some urban areas sustain high densities of frog populations [27,28], in some cases even preserving relictual populations of species that have been devastated by disease [29]. Finally, although factors such as vegetation cover have been shown to influence frog *occurrence* in urban ecosystems [30–33], there has been less work to understand how these variables interact to influence population *trajectories* (although see ref [34]). Here, we seek to address these knowledge gaps via a case study from a temperate, modern city in southeastern Australia (Canberra).

We made several *a priori* predictions as to the kinds of patterns that we expected to observe during our analysis, based on prior research in our study region and elsewhere. In particular, we did not anticipate a decreasing trend in species richness over our study period, as unlike many other locations [35,36], the disease chytridiomycosis no longer appears to be causing declines in our study region, despite previous extinctions and ongoing high prevalence [37]. However, important population-level processes such as breeding and dispersal are impeded by the loss of wetlands and terrestrial habitat that occurs during urbanization [30]. Our metric of urban land cover–the percentage cover of bare ground within the landscape surrounding each survey location–is a proxy for several such processes. Therefore, we expected to observe faster declines of some frog species in areas of high urbanization; i.e. that there would be an interaction between urban land cover and survey year as predictors of frog occurrence. We also anticipated that several hydrological wetland attributes–including their size, water flow rate, and spatial arrangement–would influence their probability of occupation by frog species, and so controlled for these effects by including them as covariates in our analyses.

## Materials and Methods

### Ethics statement

This field study was established under the direction of the Australian Capital Territory (ACT) Government Environment and Sustainable Development Directorate and the University of Canberra. Sampling was strictly observation-only and no animal handling was involved. The study was conducted on public and privately-owned land, and access permission was granted by landowners prior to establishing the field sites.

### Study area and species

**Fig 1:**
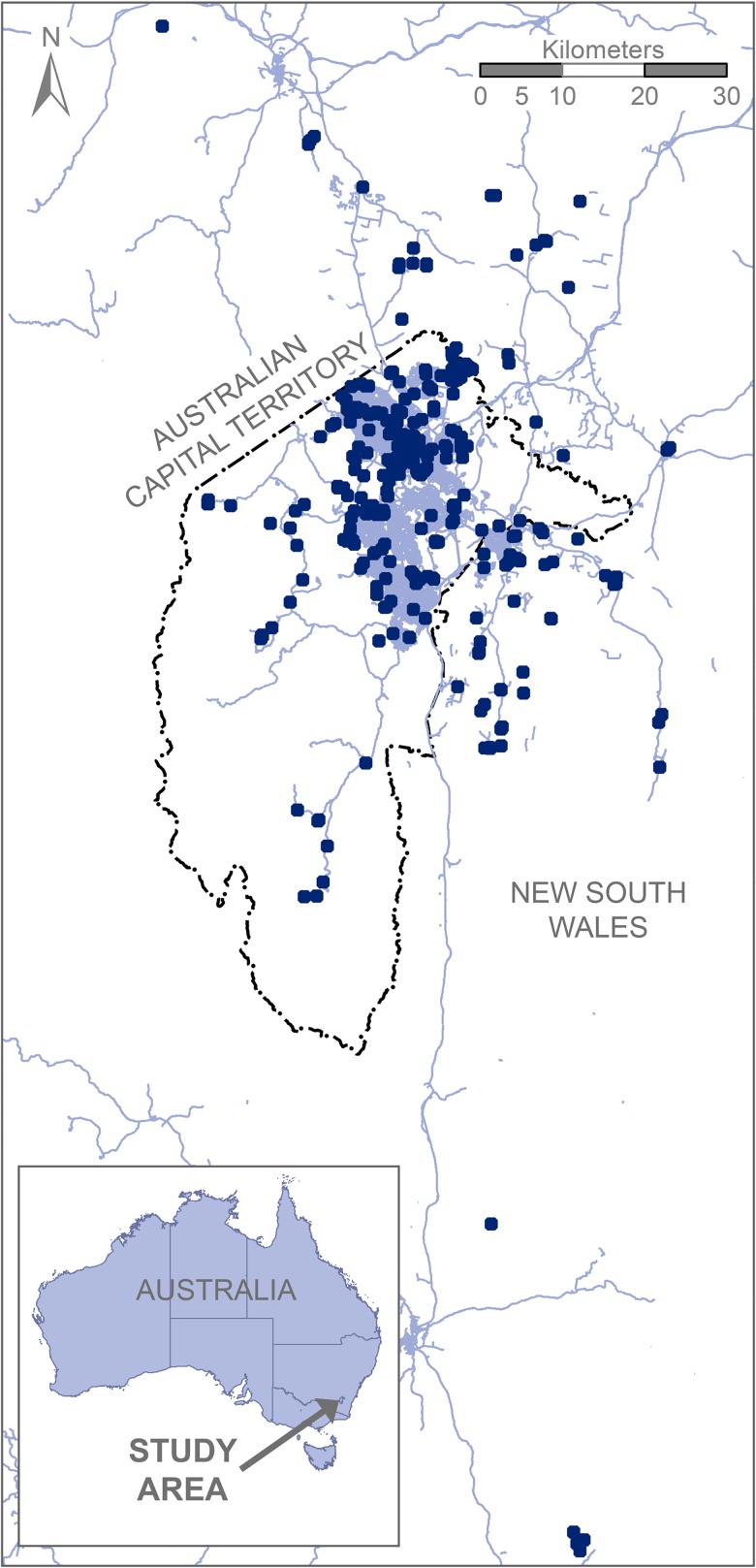
Map of Frogwatch survey locations. Sealed roads are solid blue lines.

Our study was undertaken in the Canberra region of the Australian Capital Territory (ACT) and included surrounding areas of New South Wales (NSW), southeastern Australia (Fig 1). This relatively small geographic area (~2,500 square kilometres, 37.3’ S, 149.1’ E) contains a population of ~380,000 people, and has low precipitation relative to adjacent coastal and mountain regions (mean annual precipitation = ~690mm). Importantly, our study began during the severe Australian Millennium drought, which ended in 2009 [38]. Consequently, our results must be interpreted considering the potential for climate-based fluctuations in water availability–and therefore in frog populations–over our study period.

The Canberra region provides a particularly interesting context for investigating processes that drive change in urban frog populations. The emergence of chytridiomycosis in the ACT during the 1980s is the most likely cause of the extinction of three species and significant declines in two others [37,39,40], one of which is now re-expanding [37]. This suggests that frog populations in this region are likely to be in flux, increasing the need for reliable long-term population-level data to evaluate trends and ongoing threatening processes. Further, Canberra is a relatively young, low density, but rapidly expanding city with an active policy of maintaining shared green space via the provision of parks and nature reserves [11]. This practice should be beneficial to biodiversity in general, but may have different impacts on different plant and animal taxa. Finally, there is a great deal of community involvement in citizen science initiatives that regularly monitor bird and frog populations throughout the Canberra region (e.g. see [41]), providing a valuable source of information on population trajectories.

Eighteen species of frogs are known from the ACT and nearby regions [42]; however only ten species currently occur in or near urban areas. We had sufficient data to investigate the occurrence of eight species from three families (Hylidae: *Litoria peronii* and *Litoria verreauxii*; Limnodynastidae: *Limnodynastes dumerilii*, *Limnodynastes peronii* and *Limnodynastes tasmaniensis*; Myobatrachidae: *Crinia signifiera*, *Crinia parinsignifera* and *Uperoleia laevigata*). The remaining two species (*Litoria aura* and *Neobatrachus sudelli*) were exceptionally rare in our dataset, and so we restricted our investigation of species richness to the same eight species discussed above.

### The Frogwatch dataset

We built our models of frog species occurrence and richness using data generated by the Frogwatch ACT and Region Program (hereafter referred to as ‘Frogwatch’), a citizen science initiative that has been run by the Ginninderra Catchment Group since 2002. Sites were selected by volunteers, and consisted of a single surveyable portion of the wetland edge. This definition ensures compatibility between vegetation and frog attributes, but also means that our data represents a subset of the total habitat available in large lakes or rivers. Fieldwork in the Frogwatch program uses auditory sampling to record occurrence of calling males, following a standardized procedure. Surveys may be taken at any time during October each year (during the austral spring when most species are breeding), although the majority of surveys occur during the designated ‘census week’ (beginning from the third Sunday in October). All volunteers make an initial site visit during the day to record attributes of the pond or waterway that they are investigating. This is followed by an evening visit (between sunset and 10pm) to identify calling frog species, along with associated weather and water and air temperature. Surveys include taking audio recordings of the frog chorus which are validated by the Frogwatch coordinator (AMH) prior to inclusion in the dataset.

For the purpose of this analysis, we used data collected between 2002 and 2014 (inclusive), incorporating 3,967 visits to 486 unique sites (Fig 1). However, we removed 148 sites as they were visited on <3 occasions during the study period, which can increase the difficulty of fitting mixed models in analyses such as ours [43]. We excluded a further 18 sites because they lacked data on one or more site-level covariates. This left us with data from 3,633 visits to 320 sites for further analysis. Sites were visited an average of 11.3 times over the 13 years (median = 8 visits), in an average of 5.2 of the 13 survey years, while 28 sites (9%) were visited in ten or more years.

### Site-level predictor variables

We investigated the effects of seven site-level predictor variables, derived from three distinct sources. The first was a set of general hydrological or vegetation attributes for each site, which we quantified using volunteer responses to site survey questionnaires. The first of these was a binary variable (waterbody type) that described whether a site contained still or flowing water. We chose the most common response amongst all visitors to each site as the ‘correct’ value for this variable. The second was a continuous variable describing the estimated size of each waterbody, calculated as the mean of all reported, log_10_-transformed waterbody areas (in metres squared) for each site. Finally, we used volunteer descriptions of terrestrial and aquatic vegetation to estimate the quality of local vegetation at each site. Aquatic vegetation was scored as zero if ≥ 50% of visitors to that site reported no vegetation, one if only algae was reported, two if aquatic or emergent vegetation was present, and three if both aquatic and emergent vegetation were reported. Terrestrial vegetation was recorded as the number of reported vegetation strata (under-, mid- and over-storey) at each site. Our final variable (local vegetation) summed these two metrics to give a continuous, numerical variable describing vegetation extent. For all of the volunteer-derived covariates, high variability in volunteer responses forced us to assume no change in these variables over time at each site.

Our second set of variables were landscape-wide surface cover attributes, which we extracted from the annual Terra MODIS Vegetation Continuous Fields dataset (which has a resolution of 250 metres [44]). For each Frogwatch site, we calculated the average values of two variables (described below) from all pixels within a buffer of 500 metre radius, excluding any pixels that were recorded as water. The first variable, representing urbanization, gave the proportional area of bare ground, which we use as a proxy of percent urban land cover. Bare ground was a useful proxy for urbanization because it was strongly correlated with a more direct measure of urban density (i.e. road length within a 500 metre buffer) in our study region, and was available for all study years. Although this variable was continuously distributed (ranging from 1% to 35% bare ground cover), we refer to sites with extreme values of this variable as either ‘rural’ (tenth percentile) or ‘urban’ (ninetieth percentile). We categorized sites in this way for clarity; but as Canberra is a low density city by global standards [1], readers should not consider ‘urban’ sites in our analysis as comparable to high density urban locations in other cities. The second variable gave the percent cover of canopy vegetation. As both variables were skewed towards very low values, we logit-transformed both covariates prior to including them in our analysis. Unlike our volunteer-derived variables, we used annual data for MODIS-derived variables, and so our analysis investigates the effect of urbanization and canopy cover while allowing both values to change at each site over the study period.

The third set of variables was included to account for potential statistical anomalies in our dataset. We derived a factorial variable describing the identity of each site for use as a random effect. We also calculated a spatial autocovariate for each response variable using the autocov_dist function from the spdep R package [45], to account for spatial structuring of species richness or occurrence patterns. In datasets where survey effort is inconsistent, a variable describing the number of species observed per visit (the ‘list length’) is often informative [46]; however this was not necessary in our case as survey effort per visit is standardised in the Frogwatch program.

Prior to our analysis, we scaled all continuous predictor variables (i.e. local vegetation, % urban, % canopy and all spatial autocovariates) to a mean of zero and a standard deviation of one. As a result, all coefficients presented can be compared between each other, or between models for different species.

### Statistical analysis

We used generalized linear mixed models (GLMMs) to model variation in species richness and occurrence, including a list of site identifiers as a random effect to account for non-independence caused by repeat visits to each site [43]. We were unable to fit models that account for variable detection rates (e.g. [47]) because sites were not visited consistently each year, and because some species were too rare to parameterize such a model. Consequently, our approach confounds occupancy and detection, meaning that we are unable to identify what proportion of change in observation rates resulted from change in species distributions, rather than changes in detectability. Further, interpreting our results purely in terms of occupancy rates requires the assumption that detection does not vary systematically in relation to our predictor variables, which may be unrealistic. However, simulations have shown [48] that the data collection, cleaning and analysis methods that we used–including consistent effort per visit, removal of rarely visited sites from the dataset, and accounting for site-specific observation probabilities–results in a model that has low risk of systematic bias, and only marginally increased type I error (i.e. mistakenly identifying strong effects that are not present) without an increase in type II error (likelihood of missing strong effects when they are present). We therefore report our results without further reference to detection probabilities, while acknowledging that such research would be informative.

We investigated occupancy of each species using a binomial mixed model with a logit link, while for species richness we used a Poisson model with a log link. Clearly, the results of the species richness model will be strongly related to the sum of the eight individual species models that we also present; however we include it in our analysis for completeness, and because richness is a common summary statistic of use in the management of frog populations in our study region. We used these models to test a range of hypotheses regarding the influence of urbanization and vegetation on occurrence or trajectory of frog populations using a model selection approach (Table 1). We calculated all models using the lme4 package [49] in the R statistical program [50], and selected the ‘best’ model as that with the lowest AIC_c_ [51], while treating all models within two AIC_c_ of the best model as plausible.

**Table 1:** Outline of all models tested for relative fit to frog occurrence or richness patterns.

Model	Formula	Hypothesis
1	Response ~ Vegetation + Size + Type + Autocovariate + (1 | Site)	1
2	+ % Canopy	1
3	+ % Urban	1
4	+ Year	1
5	+ % Urban + % Canopy	1
6	+ % Urban + Year	1
7	+ % Canopy + Year	1
8	+ % Urban + % Canopy + Year	1
9	+ % Urban * Year	2
10	+ % Urban * Vegetation	3
11	+ % Urban * % Canopy	3
12	+ % Urban * Year * Vegetation	4
13	+ % Urban * Year * % Canopy	4
14	+ % Urban * Year * % Canopy * Vegetation	4

All models include all terms in model 1, plus those terms listed in the ‘formula’ column for that model. Hypotheses: 1. Occurrence or richness of frog species is affected by waterbody attributes, and/or is changing over time; 2. The effect of time on frog occurrence or richness varies across the urbanization gradient; 3. The effect of time on frog occurrence or richness varies across the urbanization gradient; 4. The effect of time on frog occurrence or richness is influenced by urbanization and one or more vegetation-related attributes.

## Results

Model selection by AIC_c_ showed that complex models were nearly always selected to describe frog population trajectories in our study region, with models that included interactions being strongly weighted for six of eight species, as well as for species richness (Table 2). This constitutes strong support for the hypothesis that frog population trajectories vary between sites with differing urban land cover and vegetation attributes. However, we were unable to conclusively determine a final model for half of our eight species. Model selection was particularly equivocal for two species from the genus *Limnodynastes*; namely *Lim*. *dumerilii* (four models with ΔAIC_c_ <2) and *Lim*. *peronii* (five models with ΔAIC_c_ <2). We were also unable to discriminate between very similar models for *C*. *signifera* and *Lim*. *tasmaniensis* (Table 2).

**Table 2:** Change in AIC_c_ from the ‘best’ model of frog species occurrence or richness.

	Model													
Response	1	2	3	4	5	6	7	8	9	10	11	12	13	14
*C*. *parinsignifera*	28.04	16.12	29.60	24.89	11.95	24.07	16.26	8.75	25.43	26.20	5.75	20.74	3.30	**0.00**
*C*. *signifera*	74.20	75.00	10.47	57.09	7.75	9.47	59.01	6.30	11.48	6.21	6.35	6.99	**0.00**	**0.05**
*Lim*. *dumerilii*	7.97	8.48	**0.00**	9.97	**1.96**	**0.94**	10.44	2.95	2.01	**1.78**	3.95	2.28	5.35	7.03
*Lim*. *peronii*	2.80	2.07	2.81	**0.28**	3.34	**1.61**	**0.44**	2.31	**0.04**	3.36	2.37	**0.00**	**0.77**	6.25
*Lim*. *tasmaniensis*	18.31	15.17	18.61	19.53	11.21	18.55	17.01	11.78	19.11	18.28	**1.58**	19.42	**0.00**	5.61
*Lit*. *peronii*	32.83	33.87	20.21	34.71	21.99	20.94	35.86	22.80	22.52	19.21	19.03	16.56	**0.00**	**0.73**
*Lit*. *verreauxii*	36.19	38.01	37.91	11.34	39.39	11.40	9.56	11.00	13.21	28.91	39.96	**0.00**	15.52	5.03
*U*. *laevigata*	73.18	75.03	29.97	48.68	27.21	22.67	50.20	18.50	24.23	30.45	24.11	28.16	**0.00**	10.89
Spp. Richness	48.54	49.91	19.37	43.38	9.99	20.87	43.25	10.90	21.45	11.47	8.94	13.10	5.06	**0.00**

All models with AIC_c_ <2 shown in bold, with ‘top’ model for each species underlined. Note that model formulae are given in Table 1.

**Fig 2:**
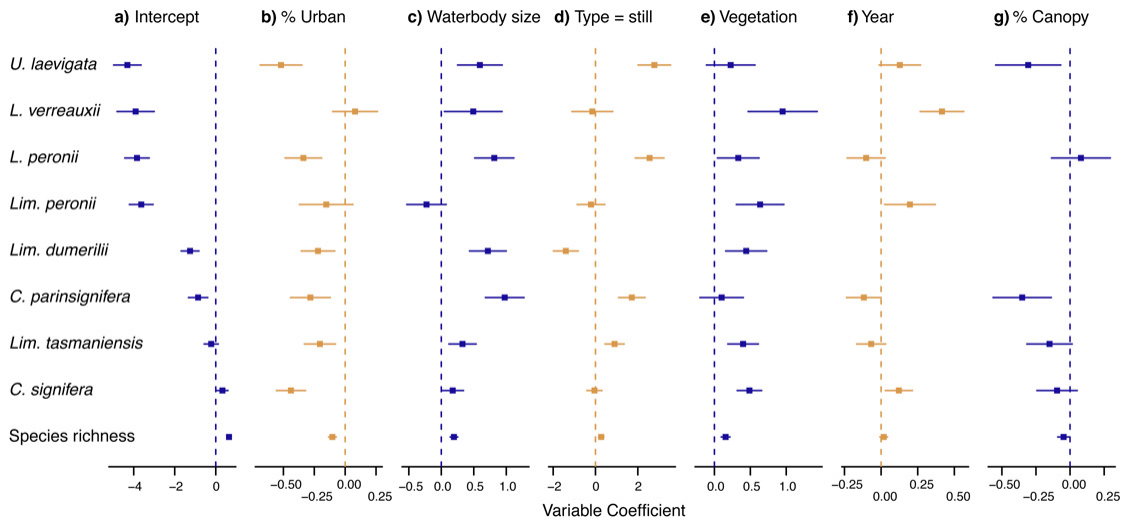
Coefficient estimates from final models for each species. Plots are ordered by mean effect size (calculated as the coefficient divided by the standard error of that coefficient) across all models, with highest effect size on the left and lowest on the right. Species are shown in increasing order of prevalence (top to bottom). Colours are to distinguish between plots only, and do not have any inherent meaning. Only variables included in the final model are shown (hence missing values in panel f), while interactions are not shown. Axes vary in scale between subplots.

We found strong, negative effects of urban land cover on the occurrence of six of the eight species that we studied (i.e. all species except *Lim*. *peronii* and *Lit*. *verreauxii*), as well as lower species richness in urban than rural wetlands (Fig 2). Further, large wetlands were more likely to contain individuals from all species except *Lim*. *peronii*, while half of the eight species we studied were more likely to be found in still than flowing water (in contrast, *Lim*. *dumerilii* was less likely to occur in still water). Finally, site-level vegetation was positively related to the likelihood of observing the majority of frog species, while percentage canopy cover within 500m had either no effect or a moderate negative association with frog occurrence.

**Fig 3:**
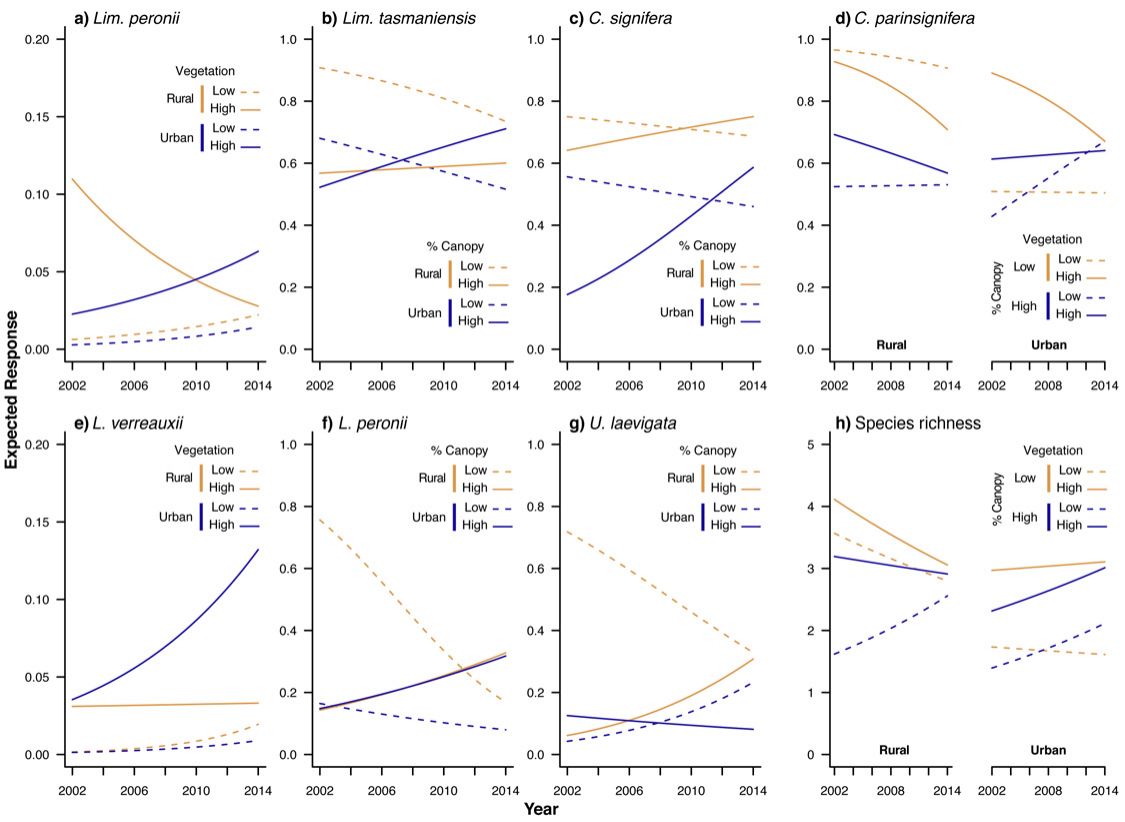
Modelled frog occurrence and species richness over time in the ACT region.

Although most frog species were less likely to be found in urban than rural wetlands, our model selection results (Table 2) suggested that the effect of urbanization on population trajectories was complex, and interacted with site-level and landscape-wide vegetation patterns. Plotting results from all ‘best’ models that included an effect of time (i.e. for all species except *Lim*. *dumerilii*) supported this expectation, showing that none of the species that we studied have declined consistently within urban areas (Fig 3). Instead, the fastest declines in our study region appear to be occurring in rural areas. For example, *Lit*. *peronii* (Fig 3F) and *U*. *laevigata* (Fig 3G) are both declining in rural areas with low canopy cover, but increasing in prevalence in rural areas with high canopy cover. Similarly, *Lim*. *peronii* (Fig 3A) appears to be declining only in rural wetlands with high site-level vegetation; but this is the result of a model with low weight and should not be considered conclusive. In contrast, every species showed increases in prevalence in at least a subset of urban areas, although these increases were generally small and from a low baseline. Overall, therefore, our results show that frog trajectories in urban areas are highly conditional on the amount of vegetation at the pond and in the surrounding landscape.

## Discussion

In this study, we investigated trends in the composition and richness of frog assemblages from an urban landscape in southeastern Australia, using a citizen science dataset of high spatial and temporal resolution. Consequently, our results provide a comprehensive insight to the viability of frog populations in our study region, showing that several species occur at lower incidence in urban areas. However, urban populations were relatively stable over the 13 years of observation, suggesting that many frog species may be able to persist in these locations in the medium- to long- term (albeit at a low proportion of available wetlands; see Fig 3). Even more encouragingly, the majority of species in our study were increasing in prevalence in well-vegetated urban wetlands. This suggests that local conservation and remediation activities might be capable of sustaining both urban and rural frog populations. We further discuss these results and their implications in the remainder of our paper.

### Influence of urban density on frog assemblages

Our results clearly show that urban areas within the Canberra region support lower richness and incidence of frog species than their rural equivalents. In particular, six of the eight species that we studied (i.e. all except *Lim*. *peronii* and *Lit*. *verreauxii*) showed strong, negative effects of urban density on their probability of occurrence (Fig 2). While the precise mechanism by which urbanization influences frog populations remains unclear, it is likely that urbanization influences frog dispersal rates, as well as the quality of aquatic habitats for frog survival and reproduction [52]. For example, urban environments strongly reduce the rate of frogs’ inter-pond movements [53], and may increase the probability that frogs will suffer predation from pets [54], reducing the chance that local populations can be maintained via colonization (and thereby reducing the overall occupancy rate; see [55]). This supports earlier research showing that urban wetlands display a series of correlated attributes that reduce habitat suitability for many frog species [30].

Despite lower richness and incidence of frog species in urbanized areas, our results indicate that urban sites can sustain some frog species through time [52]. In particular, we found no evidence of ongoing declines in urban areas for any species (Fig 3). This finding supports research from other locations showing persistence of frog populations in cities that are older than Canberra by several decades [30] or even centuries [56], and suggests that urbanization may not induce the long extinction lags that have been recorded in other taxonomic groups such as plants [16] or butterflies [15]. However, the lower incidence and diversity of frog species in urban areas indicates that urban-sensitive species may have already gone extinct in highly urbanised sites. Our results therefore suggest that increasing urbanization over time–either by expansion of the urban boundary, or increasing density of existing suburbs–is likely to be associated with further declines in frog populations in our study region.

Contrary to our predictions, we found that several frog species (*Lit*. *peronii*, *U*. *laevigata* and possibly *Lim*. *peronii*) appear to have declined rapidly in some rural wetlands around Canberra (Fig 3D and 3E). Why declines should be largely restricted to rural areas during our study period, and to these species in particular, is unclear. One possibility is that–as our data are collected in the same month each year–changes in breeding phenology could appear as declines in our analysis, suggesting a potential influence of climate change on our results [57]. Alternatively, undocumented changes to rural ponds may have occurred near Canberra during our study period. For example, changes to agricultural practices such as fertilizer use or stocking rates could potentially have influenced occupancy rates of these two species (see [58]). Finally–and in common with all forms of statistical investigation–our results are subject to error, and so some caution is required in interpreting the precise rate and location of population declines that we report. Nonetheless, identifying the relative importance of these processes for driving changes in frog assemblages should be a priority for future research, to allow targeted activities to prevent any such declines.

Our finding that the effect of urbanization on frog populations is mediated by vegetation is potentially important for the management and rehabilitation of urban wetlands. In particular, our results suggest that increases in landscape-wide canopy cover (for *Lim*. *tasmaniensis*, *C*. *signifera and Lit*. *peronii*), site-level vegetation cover (for *Lim*. *peronii*), or both (for *C*. *parinsignifera*), can reduce or even reverse population declines. However, two important caveats to these conclusions should be made. First, our results are correlative and should not be confused with experimental results; it is possible that some unmeasured attribute of urban wetlands is responsible for the changes in frog populations that we have documented (although see [59]). Second, the effects of each vegetation variable varied enormously between species, between locations, and in relation to their respective effects on occupancy rates or population trajectories. For example, high canopy cover was associated with increases in prevalence of *U*. *laevigata* in rural areas, but led to decreased prevalence in urban areas (Fig 3G). Similarly, sites with low canopy cover and low site-level vegetation had the highest occupancy rates of *C*. *parinsignifera* in rural areas, but the lowest occupancy rates in urban areas (Fig 3D). Therefore, planners seeking to facilitate the survival of frog populations in our study region should consider the habitat requirements of those species they most wish to benefit, as any single intervention will benefit some species more than others.

Overall, our results suggest that large, still, well-vegetated wetlands in rural areas are most likely to sustain diverse frog assemblages in our study region. In particular, high levels of aquatic vegetation always had a positive effect on species occurrence, and for five of our study species (*C*. *signifera*, *Lit*. *verreauxii*, and all three *Limnodynastes* spp.) this effect was stronger than the negative effect of urbanization. This is encouraging, as it suggests that prevalence of frogs in urban areas may be increased by revegetation of frog breeding sites. Our work also contradicts evidence [60] that permanent flooding is largely detrimental to frog populations (Fig 2), possibly because most large, still wetlands in our study region are artificial and so lack diverse assemblages of predatory fish. In combination, these results suggest that comparably straightforward management interventions (such as stock exclusion in rural areas, and revegetation in urban wetlands) could provide large benefits to frog populations across our study region [61].

### Future directions

Citizen science initiatives are becoming increasingly common in a number of disciplines, and our results highlight both the strengths and potential pitfalls of such datasets (see also [20]). In particular, citizen science programs typically provide good data on common species [62], which are valuable for assessing long-term changes in biodiversity resulting from climate change or land use shifts. Long-term datasets–and long-term public engagement with nature–also help to counteract the ‘shifting baselines’ problem, whereby management goals become less stringent with time due to altered perception of the ‘ideal’ ecosystem state [63]. In contrast, a widely recognized limitation of citizen science is that there is limited scope to customize monitoring to focus on particular locations, species, or trends [64]. For example, species of conservation concern often require specialised, intensive monitoring programs [65], and so their population status can be difficult to assess using citizen-based approaches. Similarly, attributes of ecosystems that are undetectable through passive observation require specialist assessment, of which an important example in our study is the distribution and impact of chytrid fungus [37]. Consequently, it is important when assessing the value of citizen science initiatives to consider both the strengths and weaknesses of this method of monitoring biodiversity.

A particular issue in our investigation was that several statistical attributes of the Frogwatch dataset may have limited our capacity to infer trends in frog populations. For example, we identified declines of two species in rural areas, but most observations were in areas of high urban land cover (Fig 1), potentially reducing our understanding of the magnitude and severity of these declines. This is indicative of a broader issue with studies such as ours, namely that statistical power to detect long-term, shallow declines in animal populations is typically low [66], and can only be achieved through monitoring over longer periods than Frogwatch has achieved to date [67]. Further, our process of removing rarely visited sites from the dataset prior to analysis is known to reduce statistical power for detecting trends (i.e. to increase type II error [48]). Finally, the structure of our data necessitated that we confound occupancy and detection in our analysis, with the result that we are unable to report estimates of absolute occupancy rates. These considerations are important from a management perspective because they reduce our capacity to identify and intervene in population declines. Fortunately, the problems that we have outlined above could be substantially addressed by focussing available research effort on a smaller number of sites, while ensuring that each site was consistently visited on more than one occasion per year. Although direction of research effort is difficult in any volunteer program [64], this strategy has now been adopted as a guiding principle for future work in the ACT Frogwatch program.

In addition to statistical issues, the distribution of research effort in the Frogwatch program also biased our conclusions towards species that were tolerant of urban environments. This meant that we were unable to reach any conclusions regarding the trajectory of several species in our study region, including any species that were restricted to riverine or montane environments. For example, we were able to verify the documented expansion of *Lit*. *verreauxii* in our study region over recent years (see Fig 3E, [37]), but were unable to test anecdotal suggestions that *Litoria latopalmata* may also be expanding (W. Osborne, pers. obs.). Similarly, *Neobatrachus sudelli* displays explosive breeding following high rainfall events, and so is rarely detected by Frogwatch volunteers, reducing our knowledge of the distribution and trajectory of this species. Despite these knowledge gaps, we do not advocate the expansion of the existing program to accommodate a wider range of survey sites and seasons, as this strategy would be inconsistent with our call for more targeted data collection at a smaller number of sites (see above). Instead, we suggest that if there is a strong need for data on cryptic or rarely detected species, then that data should be collected by professional ecologists as part of a targeted monitoring program [58,64].

## Conclusions

Using a long-term citizen science dataset, we have quantified the association between wetland attributes and the distribution and trajectory of frog populations in an urban landscape. Our results show that the effects of urbanization are mediated by wetland vegetation structure and canopy cover in the surrounding landscape. Frog declines in the Canberra region are largest in the rural hinterland, with urban frog populations persisting at a comparatively small proportion of available wetlands (see also [68]). However, there was little evidence of widespread declines in urban wetlands, with populations of some species marginally increasing in prevalence in urban areas with suitable site-level or landscape-wide vegetation. These findings provide robust support for landscape planning and management to sustain diverse frog assemblages in our study region. We suggest that the greatest opportunity for frog conservation in our study region is through the conservation and restoration of rural wetlands to arrest current declines. In comparison, revegetation of urban wetlands has strong potential as a method to facilitate the re-expansion of urban-sensitive species.
